# Recent advances in adoptive cancer immunotherapy in china

**DOI:** 10.1186/2051-1426-3-S2-P64

**Published:** 2015-11-04

**Authors:** Juhua Zhou

**Affiliations:** 1 Ludong University, Lexington, SC, USA

## Background

Cytokine-induced killer (CIK) cells have been extensively used in treatment of cancer patients in China. Clinical trials demonstrated that there were markedly decreased tumor nodules in size in a patient with advanced pancreatic adenocarcinoma following four cycles of CIK cell infusion and thus the patient had a longer progression-free survival. Moreover, immunotherapy with CIK cells in combination with chemotherapy had more potential benefits including longer progression-free survival and overall survival in patients with cancer such as advanced gastric cancer and non-small-cell lung cancer as compared with chemotherapy alone. In addition, dendritic cell (DC)-CIK cells have increased proliferation ability, cytokine secretion and anti-tumor activity as compared with CIK cells alone.

## Results

A clinical trial showed that immunotherapy with tumor lysate-pulsed DC-CIK cells could significantly increase overall survival rates than no treatment control in renal cancer carcinoma patients. Thus, nowadays, DC-CIK cells have also been extensively used in treatment of cancer patients in China although the efficacy and mechanisms of adoptive cell transfer therapy with DC-CIK cells remains to be determined. In one word, the accumulation of basic researches and clinical studies related to cancer immunotherapy with CIK and DC-CIK cells has confirmed their safety and feasibility in treating malignant diseases. However, at present, there are still no uniform criteria or large-scale preparations of CIK and DC-CIK cells and the overall clinical response is difficult to evaluate due to lack of practical and appropriate criteria.

## Conclusions

Large-scale, controlled, grouped, and multi-center clinical trials on CIK and DC-CIK cell-based immunotherapy should be conducted in the near future.

